# Peyronie's Reconstruction for Maximum Length and Girth Gain: Geometrical Principles

**DOI:** 10.1155/2008/205739

**Published:** 2008-12-03

**Authors:** Paulo H. Egydio, Salvatore Sansalone

**Affiliations:** ^1^Urology Institute, Rua Iguatemi 192 Cj. 42, São Paulo 01451-010, Brazil; ^2^Policlínico di Tor Vergata, Università di Roma, Viale Oxford, 81, 00133 Roma, Italy

## Abstract

Peyronie's disease has been associated with penile shortening and some degree of erectile dysfunction. Surgical reconstruction should be based on giving a functional penis, that is, rectifying the penis with rigidity enough to make the sexual intercourse. The procedure should be discussed preoperatively in terms of length and girth reconstruction in order to improve patient satisfaction. The tunical reconstruction for maximum penile length and girth restoration should be based on the maximum length of the dissected neurovascular bundle possible and the application of geometrical principles to define the precise site and size of tunical incision and grafting procedure. As penile rectification and rigidity are required to achieve complete functional restoration of the penis and 20 to 54% of patients experience associated erectile dysfunction, penile straightening alone may not be enough to provide complete functional restoration. Therefore, phosphodiesterase inhibitors, self-injection, or penile prosthesis may need to be added in some cases.

## 1. INTRODUCTION

Peyronie's disease (PD) is characterized by scar tissue development in the tunica albuginea, which makes it less elastic, causing penile deformity, and is invariably associated with a decrease in penile functional length. The condition has an impact on quality of life, and a significant psychological effect on 77% of patients [1].

Surgical decision is made after clinical treatment failure, when penile deformity (curvature, narrowing, or indentation) and plaques are completely stabilized, and pain has been absent for at least 6 months, provided that the patient experiences functional penile inadequacy.

Association between PD and erectile dysfunction (ED) is seen in 20% to 54% of cases [2]. Careful assessment of this associated condition is a key to correctly determine the need for surgery and to ensure the success rate of reconstruction procedures.

Penile deformity is consistently associated with functional length reduction, since the penis curves because one of its sides has lost more elasticity than the other.

A curved penis has a short and a long side. If an attempt is made to straighten it by shortening the longer side, this may not be satisfactory for the patient, because a decrease in final penile length may result. This decrease is proportional to the degree of penile curvature. It is possible during pharmacologically induced erection to estimate the penile size if the long side is going to be reduced and it is recommended to ask the patient whether that length will be enough to make him satisfied.

Therefore, for selected cases, surgical treatment should focus on functional penile-length restoration. Lengthening the shorter side is the alternative that provides maximum gain in penile length.

Surgical treatment is aimed at providing good penile function (i.e., rectification as well as adequate length and enough rigidity to enable healthy sexual activity). The geometrical technique is the most precise procedure to lengthen the short side, thereby recovering the length lost by scarring. Penile straightening is indicated for patients with normal spontaneous erection or erectile dysfunction that responds to medication, whereas those with untreatable erectile dysfunction requiring penile prosthesis [3, 4] can have it implanted during the reconstructive procedure.

The size of the prosthesis is compatible with the longer side, as the shorter side is the one to be lengthened. Maximum length restoration was possible and limited by the length of the dissected neurovascular bundle [5].

## 2. MATERIALS AND METHODS

### 2.1. Preoperative Evaluation

#### 2.1.1. Sexual and Medical History

Preoperative evaluation should include complete clinical history as well as assessment of comorbidities, such as diabetes, heart/vascular/coronary conditions, arterial hypertension, smoking, alcohol consumption, signs and symptoms of hypogonadism, and regular medications, which may affect erection.

A detailed history should be obtained on associated erectile dysfunction, either prior to or concomitant with Peyronie's disease, as well as risk factors contributing to the development of the condition, such as sexual partner's lubrication status, achievement of an erection that continues until ejaculation, premature or late ejaculation, or inadequate habits that may cause injury to the tunica albuginea. A history of phosphodiesterase-5 (PDE5) inhibitor use is a key to establishing the presence of associated erectile dysfunction, as well as the response of this condition to the medication, the patient's tolerance to its side effects, and his compliance with treatment.

PD is consistently associated with shorter penile length. Some patients experience symmetric loss of elasticity, with little or no deformity. In such cases, a decrease in penile length may be the sole complaint.

#### 2.1.2. Assessment of Penile Deformity, Rigidity, Vascular Status, and Arterial Anomalies

A complete evaluation is essential in cases of sexual inadequacy with possible surgical indication. Patients with erectile dysfunction may need specific treatment, and assessment of their response to treatment before surgery is considered as a therapeutic option.

For deformity assessment, physical examination of a flaccid penis may reveal a palpable thickened tunica. Penile size may be determined by pulling the glans penis forward and upward to the position of a normal erection and asking the patient to indicate to which extent PD has shortened his penis.

Erection assessment is essential to establish surgical indication as well as the most appropriate surgical procedure. Penile tumescence, or partial rigidity, is often mistaken for erection, and the objective test of pharmacologically induced erection may change the therapeutic plan.

Rigidity assessment is performed both subjectively, as reported by the patient, and objectively, as observed by the physician after intracavernous injection (ICI) of alprostadil 10 to 20 *μ*g, which allows evaluation of penile deformity and objective rigidity, and, with Doppler ultrasound (DUS), provides essential data for vascular assessment (arterial insufficiency and/or veno-occlusive dysfunction) as well as detection and localization of collaterals between dorsal and cavernous arteries.

After ICI, the patient holds his penis in an erection position, and the ultrasound scanning of thickened areas of the tunica, associated or not with calcification, is initiated. The measurement of flow indices—peak systolic velocity (PSV), end diastolic velocity (EDV), and calculated resistive index (RI)—begins at least 5 minutes thereafter, and a correlation of these indices to penile rigidity is established. One clinical study reported 44% of arterial anomalies and 10% of distal collateral arteries between dorsal and cavernous arteries [6]. In another retrospective study, vascular status was correlated to the type of penile deformity, demonstrating a relationship between type of curvature and penile hemodynamics [7].

Evaluation of patient's and partner's satisfaction and long-term results after surgical treatment for Peyronie's disease has shown that PSV values of 35 cm/s or above and RI higher than 0.9 were considered as parameters for a normal penile vascular system. EDV values above 5 cm/s were considered diagnostic for veno-occlusive dysfunction [2].

Information on penile arterial anatomy may be very useful for the surgeon to select the type of surgical technique to be used. Knowledge of the existence of a collateral branch is important to safely dissect the neurovascular bundle.

Because penile size before PD is unknown, information from the patient on the perceived extent of his penile length reduction is relevant. During erection induction for deformity assessment, the patient must be asked how satisfied he would be with the length resulting from straightening his penis by diminishing the longer side, as it is being shown to him, and which would be the extent of length loss compared to his penile size before PD. Penile length reduction by PD is very likely to have occurred when more than one site of fibrosis is seen, or when there is fibrosis on opposite sides. However, even if a thickened tunica cannot be palpated, longer-side reduction is not ruled out, since microstructural changes are enough to decrease the elasticity of the tunica [8]. There are patients with penile curvature and no palpable thickened tunica who undergo surgery. Penile deformity, not the plaque, is the main complaint of a PD patient. Surgery should focus on deformity correction rather than on plaques.

During or shortly after DUS, penile rigidity is objectively compared to self-reported rigidity. This allows more objective assessment of rigidity. If it is lower with the test, both crura penis are pressed to maximum rigidity to assess penile deformity, which will be apparent with maximum rigidity, while the other hand assesses axial rigidity by pressing on the glans to mimic a penetration attempt. If deformity is not pronounced and with good rigidity allows good axial stability, providing penile functionality, surgical treatment may not be indicated. A good erectile response to oral or injected medications may restore penetration ability in such cases.

Soon after this assessment, the patient is asked to palpate his penis and, by progressively relieving pressure on crura, to report the extent of rigidity, he observes in an ideal setting of sexual stimulation. The physician is thereby provided with an objective evaluation, and, if a rigidity deficit is proven, the patient's ED can be treated. The physician will establish what a good rigidity is, and whether this desired goal can be achieved by the patient.

#### 2.1.3. Tunical-Lengthening Procedures Based on Geometrical Principles [9–13]

**Surgical Technique**

**Figure 1:**
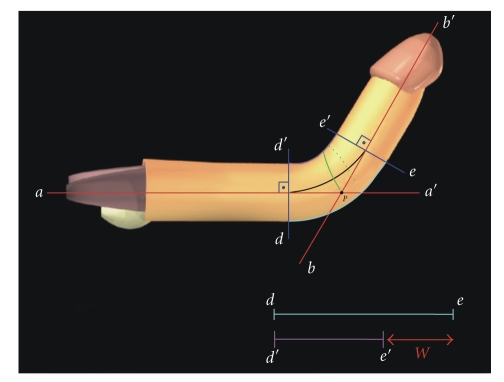
The intersection of the tangential lines to the penile axis *a*-*a*′ and *b*-*b*′ (red lines) determines the point of maximum curvature (*P*). A circumferential line is drawn (green line) from point *P* in the bisector of the angle formed by the lines *a*-*a*′ and *b*-*b*′. *W* (red arrow) is equal to the distance of the two points of the long side (*d*-*e*) minus the equivalent distance (*d*′-*e*′) in the short side of the penis (*θ* = 90°).

**Figure 2:**
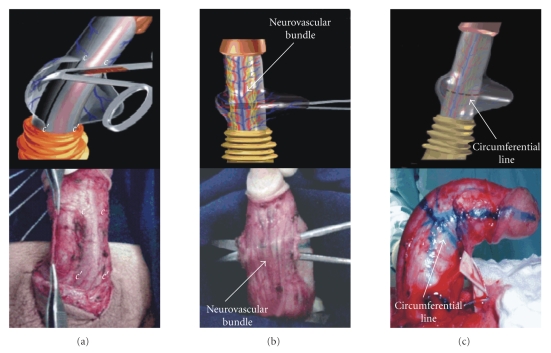
(a) Paraurethral incisions (*c*-*c*′) in Buck's fascia. (b) Dissection of Buck's fascia and the neurovascular bundle from the tunica albuginea. (c) Drawing of the circumferential line in the point of maximum curvature.

**Figure 3:**
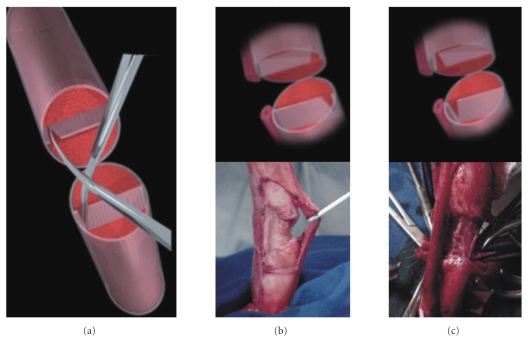
(a) Cutting of the intercavernous septum. (b) Septal cutting in cases of dorsal, dorsolateral, or lateral curvature. (c) Septal cutting in cases of ventral or ventrolateral curvature.

**Figure 4:**
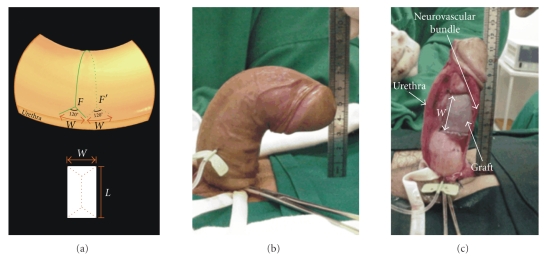
(a) Bifurcation of the transverse incision and the correspondent defects in the tunica albuginea in cases of dorsal curvatures. *W* = the width of the defect. *L* = the length of the defect. *F* and *F*′ are the points from which the circular incision is forked. (b) Preoperative dorsal curvature. (c) Final result after straightening and graft suturing.

**Figure 5:**
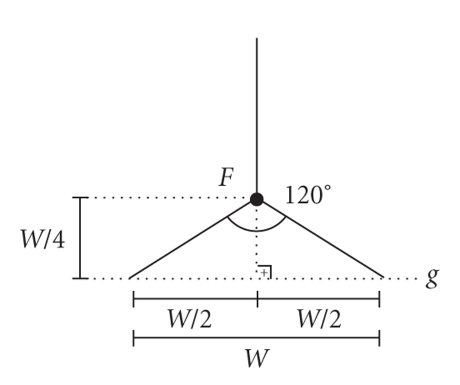
The starting point of the 120-degree bifurcation at the end of circumferential lines is established by marking a length of *W*/4 back from the intersection with the *g* line. *W* is the differences measured between the longer and shorter side of the penis that correspond to the width (*W*) of the tunica defect.

**Figure 6:**
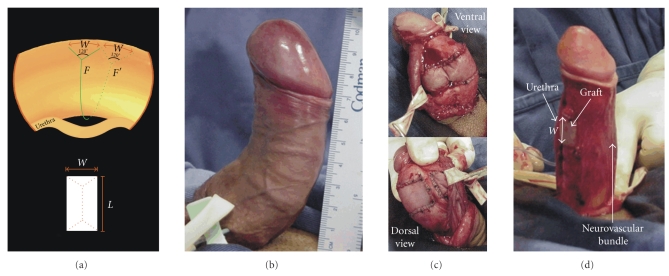
(a) Bifurcation of the transverse incision and the correspondent defects in the tunica in cases of ventral curvatures. *W* = the width of the defect. *L* = the length of the defect. *F* and *F*′ are the points from which the circular incision is forked. (b) Preoperative ventral curvature. (c) Urethral dissection. (d) Final result after straightening and graft suturing.

**Figure 7:**
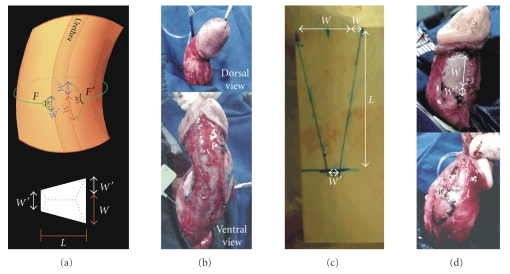
(a) Bifurcation of the transverse incision and the correspondent defects in the tunica in cases of lateral curvatures. *W* = the difference between the short and long side. *W*′ = a measure added on both side *L* = the length of the defect. *F* and *F*′ are the points from which the circular incision is forked. (b) Lateral curvature after a degloving procedure. (c) Trapezoidal graft was drawn in the pericardium. (d) Final result after straightening and graft suturing.

1. The penis is degloved after a circumcision incision. Magnifying lenses 2.5 are used for better visualization. One of the cavernous bodies is punctured by a 21 scalpel, considering that, when necessary, both cavernous bodies can be punctured to achieve full erection by saline solution injection. The use of papaverin or prostaglandin can help full erection with saline solution injection.
2. In cases of dorsal curvature, two tangential lines to the penile axis (red lines) are drawn on the proximal and distal straight segments (*a*-*a*′ and *b*-*b*′, resp.) toward the area of curvature of the erect penis (Figure 1).
3. From the point of maximum curvature (P) located at the intersection of the lines *a*-*a*′ and *b*-*b*′, a circumferential line (green line) is drawn at the bisector of the angle formed by these lines (Figure 1).
4. The point at which this circumferential line crosses the neurovascular bundle in the dorsal region and the urethra in the ventral region determines the region at which these structures must be separated from the tunica albuginea.
5. The transverse incision in the tunica will be made along this circumferential line (green line) later. Then the erection is reversed.
6. Two paraurethral incisions (*c*-*c*′) are made where the circumferential line crosses the urethra to dissect Buck's fascia and its neurovascular bundle from the tunica around the complete circumference of the penis in all types of curvature, except at the level of the urethra (Figures 2(a), 2(b)).
7. A new erection is induced and a circumferential line is drawn again, but this time on the tunica, where the circular incision will be made (Figure 2(c)).
8. Complete penile straightening is achieved by a 5-mm incision in the intercavernous septum on each side of its intersection with the transverse incision in the circumferential line (Figure 3).
9. The width (*W*) of the defect should be the same as the difference between the long and the short sides of the penis. This measurement is calculated by the distance between any two complete circumferential lines perpendicular to the penile axis drawn on the straight penile segments, that is, outside the area of curvature (before *d*-*d*′ and after *e*-*e*′) (Figure 1).
10. The difference (*W*) between *d*-*e* and *d*′-*e*′ (red arrow, Figure 1) will be the size of the defect on each side of the urethra in cases of dorsal curvature (Figures 4(a), 4(c)).
11. On the circumferential line, a length of *W*/4 away from the site where it meets the *g* line, points *F* and *F*′ (Figures 4(a) and 5) are determined to mark the start of bifurcation, which extends to either side of the *g* line at a length of *W*/2, thus generating a 120° angle (Figure 5); the resulting defect will be more simple and stable as a tripod.
12. Defect length (*L*) will be equivalent to the distance between the two paraurethral incisions for dorsal curvature, or between the two ends of the fork-shaped incision for any type of curvature passing round the short side of the erect penis (Figures 4(a), 6(a), 7(a)).
13. Once the circumferential line forked at the ends is determined, the incision is made in the tunica albuginea, producing a rectangular defect of an already known size.
14. To facilitate graft suturing, a 5-mm dissection is made between the 4 edges of the defect and the respective adjacent cavernous bodies. The graft is sutured and a new induced erection demonstrates complete penile straightening (Figure 4(c)).
15. In cases of ventral curvature, the technique is similar but with the following differences: the forking of the transverse incision is made in the dorsal region near the intercavernous septum which has its dorsal insertion maintained (Figure 6).
16. The urethra is dissected from its bed and the graft is placed between the urethra and the cavernous body (Figures 6(c) and 6(d)). A new induced erection demonstrates complete penile straightening (Figures 6(b) and 6(d)).
17. Dorsolateral curvatures with a larger dorsal component and ventrolateral curvatures with a larger ventral component are corrected by the same technique as for dorsal or ventral curvatures, respectively.
18. In cases of lateral curvature (Figure 7(a)), the defect turns out to have the shape of a trapezium instead of a rectangle as obtained in cases of dorsal and ventral curvature. The shorter side of the trapezium can vary from 0.5 to 1 cm (*W*′). The longer side (*W* + *W*′) is equal to the difference between the long and the short sides (*W*) of the penis (obtained as the other curvatures), added to the length of the smaller side of the trapezium (*W*′). The height of the trapezium (*L*) is measured as described for the other types of curvature (Figure 7(a)). Thus this procedure avoids a defect of triangular shape which would make the graft procedure more difficult (Figures 7(c) and 7(d)).
19. The graft is cut according to the measurements already made (i.e., width *W* and length *L*) but should be 1-2 mm wider and longer than the defect to provide room for the suture. However, the graft should only be this size when the material used is not likely to shrink; otherwise, a percentage for graft shrinkage should be allowed.
20. The length (*L*) of the defect should be measured with the penis erect and outside any constricted area to allow girth correction in constricted penile shaft area.
21. Buck's fascia can be sutured on place. Penile degloving is reversed and foreskin, when present, is removed to avoid postoperative swelling and/or necrosis. Circumcision incision is closed with 5.0 poliglecaprone. A light compressive dressing is applied for 7 to 10 days. Although the patient can have spontaneous erection, a 6-week period of sexual abstinence is recommended. After a 6-month follow-up, alprostadil-induced erections are used to check penile straightening in those cases a penile prosthesis has not been implanted.

### 2.2. Grafting

An ideal graft should be ready to use; available in various sizes; have good tensile strength and low potential for inflammatory reactions; infection-resistant, with minimal or no risk for disease transmission; and be cost-effective.

Several types of grafts have been used, including biologic autografts—dermis, veins, penile crura, dura mater, tunica vaginalis, fascia lata—and allografts/xenografts—cadaveric pericardium, porcine small-intestine submucosa, acellular dermis, or synthetic grafts: polytetrafluoroethylene, Dacron, or sylastic [14]. The disadvantage of using autologous grafting includes lengthening of operative time, morbidity, and scarring on the harvest site. The amount of tissue may be another limiting factor.

Hellstrom and Reddy [15] reported on using human cadaveric pericardium, as Chun et al. [16] as well as Levine and Estrada [17] did. Leungwattanakij et al. [18] compared several types of grafts in a rat model showing a low rate of inflammatory reactions with cadaveric pericardium.

Knoll [19] reported the use of porcine small-intestine submucosa (SIS) grafts for tunical substitute, with promising results. Larger-sized and more uniform patches are advantages of SIS grafts, but absorption on larger defects must be slower, requiring the use of SIS with multilayer sheet.

With the increasing use of tissue engineering, new tunica albuginea substitutes may be developed [20, 21]. Advances in this area are prominent, and grafts will be available in the future that are much more similar to the tunica albuginea, or acellular matrix that may allow the tunica to be rebuilt, whether associated with cell culture and seeding or not.

A discussion concerning the best graft often involves postoperative outcomes, although the type of relaxing incision or excision has varied. Postoperative outcomes are not solely dependent on the graft used.

A personal experience with bovine pericardium associated with plaque excision gave discouraging results. In contrast, results were promising when using the same type of graft associated with a relaxing incision procedure [9].

In another personal experience, in four cases, it was necessary to remove the pericardium graft 2.5 to 8 months after surgery (in three cases due to infection in immunocompromised patients and in one case due to absorption of graft-graft suture with dehiscence and local hematoma formation); no leakage was seen after saline-induced erection, and the operative sites were left without grafts. After the recovery period, patients still have good-quality erections and axial rigidity, and are capable of having sexual intercourse. This has shown that grafts may even be absorbable, that is, the tunica may be allowed to rebuild on the structure of the graft, provided that this allows no new blood-vessel formation, which may lead to veno-occlusive dysfunction.

It is expected that all patients have a hematoma under the graft following a grafting procedure. A personal series of 20 patients were followed for 8 months, after which the hematoma disappeared in 50% and remained as a laminar hematoma in 50%, not causing any disturbance of penile functionality based on rigidity. It is a matter of concern to maintain a large hematoma that limits the expansion of sponge cavernous tissue based on the concealed fibrotic area in the outer part of the sponge tissue. The graft is important during this period to block leakage from the sponge tissue and to maintain good penile shape.

Of the four patients who had their grafts removed and had no leakage, two maintain a permanent constriction area at the site of the removed graft, which was filled by the hematoma underlying the graft.

With the purpose of trying to maintain a minimal hematoma under the graft until blockage occurs in the outer part of the sponge cavernous tissue, a light compressive postoperative dressing is applied to be kept in place for 7 to 10 days, and the patient is started on a PDE5 inhibitor at bedtime on the 7th to 10th postoperative day, to stimulate smooth muscle relaxation, thereby expanding the cavernous tissue and compressing the hematoma as a means to help it be absorbed or transformed into a laminar shape that does not affect axial rigidity. These medications are particularly important for patients with preoperative ED, and of utmost interest to reduce the hematoma and maintain physical therapy with stimulated or reflex nocturnal erections. Early postoperative use of a vacuum device can only increase the hematoma underlying the graft, owing to negative pressure.

### 2.3. Penile Prosthesis Implantation

Patients with PD and ED that are nonresponsive to oral or injectable treatment will be candidates for penile prosthesis implantation. Depending on the type and degree of penile deformity, associated procedures (e.g., modeling, Nesbit/plication, or incision/excision as well as grafting for penile rectification and/or correction of constrictive lesions) may be necessary [22].

Rahman et al. [23] reported penile plication surgery associated with penile prosthesis. The inconvenience of this procedure is penile-length reduction. The higher the curvature degree, the greater this reduction will be.

Usta et al. [24] reported the long-term results of surgical treatment for PD, showing that penile prosthesis implantation and curvature correction with pericardium graft added no risks of complications as compared to prosthesis implantation surgery alone.

Our personal experience is that pericardium reconstruction has not increased the risk for infection and complications. This may be due to the fact that pericardial tissue, in contrast to vein and dermal grafts, needs no imbibition to survive. That is why we prefer reconstruction with pericardium grafting according to geometric principles and single incision [5, 9, 11], and concomitant implantation of malleable or inflatable prosthesis of a size compatible with the longer side, as the shorter side has been elongated.

Perovic and Djordjevic [25] described the penile disassembly technique for distal penile deformity, which allows excellent distal exposure for distal reconstruction.

From April 1999 through September 2007, 521 patients who underwent geometrical incision correction were followed up: 311 patients underwent surgical straightening without penile prosthesis implantation and 210 patients underwent reconstruction with concomitant penile prosthesis implantation (malleable prostheses for 141; inflatable two pieces for 48; and inflatable three pieces for 21 patients). Patient preference was the criteria for prosthesis type choice.

A bovine pericardium graft (Braile-Biomedica and HP-Biopróteses, SP, Brazil) was sutured into the defect and its size trimmed to 1 to 2 mm wider and longer than the tunical defect in order to include this extra size in the suturing procedure. The suture was continuous, with poliglecaprone 4.0. The greater the curvature, the greater the graft size is. Mean graft width was 3.2 ± 0.3 cm (range 2.5–4.0 cm), and mean graft length was 7.7 ± 0.4 cm (range 7.5–8.5 cm).

The mean increase in functional penile size (dependent on curvature severity) was 3.2 ± 1.7 cm (1.5–5.5 cm).

## 3. RESULTS AND DISCUSSION

311 patients underwent straightening procedure by geometrical principles and grafting without concomitant penile prosthesis implantation. Penile deformities were distributed as follows: dorsal 46% (143/311), dorsolateral 30% (93/311), lateral 12.5% (39/311), ventral 6% (19/311), and ventrolateral 5.5% (17/311). Mean penile curvature was 75 ± 15.7° (range 45–120°). The technique corrected both deformities in 15.5% (48/311) of patients with Peyronie's disease associated with penile constriction.

In four cases, it was necessary to remove the pericardium graft 2.5 to 8 months after surgery (in three cases due to infection in immunocompromised patients and in one case due to absorption of graft-graft suture with dehiscence and local hematoma formation); no leakage was seen after saline-induced erection, and the operative sites were left without grafts. After the recovery period, patients still have good-quality erections and axial rigidity, and are capable of having sexual intercourse. Follow-up by prostaglandin-induced erection of Peyronie patients who did not receive prostheses has shown penile straightening in 87% and residual curvature of up to 15° in 7% and up to 30° in 6% which does not disturb penile functioning when a good erection was obtained, either associated with PDE-5 inhibitors or not. A second surgery with penile prosthesis implantation was performed in 15% of cases whose follow-up showed deterioration of erectile function. The mean follow-up period was 45.2 ± 27.1 months (range 6–96 months). The cases with greatest curvature showed the best intraoperative gain in penile length. The gain in functional penile size was maintained in patients who kept penile straightening and was reduced by up to 0.5 cm in those who developed curvature postoperatively. The preoperative erection status was preserved in most cases whose preoperative evaluation by Doppler ultrasound showed peak systolic velocity over 40 cm/s and end-diastolic velocity under 3 cm/s. Follow-up of cases with concomitant implantation of inflatable penile prostheses showed preserved penile straightening. Satisfactory penile sensitivity was maintained.

Even the patients who had deterioration on penile rigidity and underwent a second surgical procedure for penile prosthesis implantation recovered penetration ability and re-established satisfactory sexual intercourse. Patient satisfaction is obtained when patients recover their ability for penetration while maintaining orgasm.

### 3.1. Discussion

The technique herein presented is a standardized procedure since it is based on geometrical principles and meets, as no other technique previously presented, the needs of most patients. It can be applied irrespective of the characteristics of the plaque or type of curvature caused by Peyronie's disease, either associated with concomitant penile prostheses implantation or not.

The dissection of the neurovascular bundle has been standardized for all cases by means of the two paraurethral incisions in Buck's fascia. At this level, the circumflex veins are of lesser caliber, thus permitting their cauterization, which means a smaller number of ligatures. Furthermore, when the dissection is done ventrally, these manipulations are made far from the dorsal nerves of the penis, which means a lesser risk of damaging them. Another favorable aspect is that the dissection under the bundle may be limited to the area of the curvature, allowing the possibility of its being extended if necessary. This smaller dissection of the bundle in the dorsal region minimizes the risk of lesions to the eventual collaterals between the dorsal and cavernous arteries.

The puncture of one or more of the corpora cavernosa to induce and maintain a full erection is of great importance for the correct application of these geometrical principles which define the most appropriate site for the incision in the tunica albuginea. The lines *d*-*d*′ and *e*-*e*′ may be drawn at any positions in the straight portion of the penis because the difference (*W*) between the two sides will always be the same. The crossing of the tangential lines *a*-*a*′ and *b*-*b*′ on any line parallel to the axis of the penis will always be at the bisector of the angle formed between them. The incomplete circumferential incision permits breaking all lines of force, allowing the correction of the curvature on two planes (dorsolateral or ventrolateral) by the same incision.

In lateral curvatures, a rectangular defect is created by cutting the intercavernous septum insertion in both dorsal and ventral regions. Due to the risk of erectile dysfunction that can be caused by incisions in the intercavernous septum on the dorsum and ventrum to create a rectangular tunical defect, a trapezoidal shape was chosen for the defect because, as in the other examples given, it is made by cutting the intercavernous septum at just one point (dorsal side).

The intercavernous septum may be involved in the pathogenesis of the deformity of the penis. The septal incision on both sides of the transverse incision in the shorter side of the tunica albuginea is a key to adequate lengthening of the short side and complete straightening of the penis. The traction of the penis after the incision in the tunica albuginea, the septal incision, and the dissection of the tunica albuginea from the spongy tissue of the cavernous body allowed checking whether complete straightening of the penis had been achieved. Neurovascular bundle dissection can be extended when the bundle restricts penile straightening.

A tripod-shaped bifurcation with legs 120 degrees apart from each other provides a most stable structure, allowing, according to the surgeon, better results in view of a simpler configuration of the defect in the tunica, a geometrical, more easily constructed graft shape, and a simpler suturing procedure. The bifurcations in this technique also permitted relaxation of constricted areas in the tunica and correction of associated constrictive lesions. The bifurcations in the dorsal region for ventral curvatures should not cross the intercavernous septum.

The size of the tunical defect can be calculated before tunical incision by applying the geometrical principles during a full erection, thereby allowing graft preparation even at the physician's office by induced erection.

The graft to be used may match the defect size if no graft shrinkage is likely, as is the case with pericardium grafts [9, 15]; when graft shrinkage is likely, a percentage should be added to the dimensions of the defect.

The length of the defect should be measured on an erect penis; in cases of constriction at the curvature site, it should be measured on a constriction-free site for appropriate girth restoration.

Under these circumstances, only one incision and one graft are necessary, provided that the penis shows a single point of maximum curvature (with two preferential directions only). If there are two significant curvatures at different points of the penis, two grafts may be made as described. Thus complementary plication—which not only harms the healthy side but also shortens the penis—may be avoided.

The technique herein described allows the standardization of a single tunical incision procedure that may be reproducible in multicenter studies, leading to a better understanding of the advantages and disadvantages of different types of graft material.

## 4. CONCLUSIONS

This single incision technique, applying geometrical principles, is a standardized procedure for the correction of any penile curvature, either associated with tunical constriction or not, providing maximum penile gain and girth restoration. The present technique is effective to correct all types of penile curvature, regardless of plaque characteristics. The improvement of tissue engineering techniques will contribute to the development of grafts that are increasingly close to the ideal for tunica albuginea replacement.
